# Itauba Wood Fiber (*Mezilaurus lindaviana*) and Itauba Wooden Board: A Survey on the Physical, Chemical, Thermal, and Mechanical Properties

**DOI:** 10.1021/acsomega.5c05179

**Published:** 2025-09-20

**Authors:** Heitor Luiz Ornaghi Júnior, Matheus Poletto, Matheus de Prá Andrade, Francisco Maciel Monticeli, Everton Hillig, Pierre Blanchet, Amirouche Sadaoui, Ademir José Zattera

**Affiliations:** † Postgraduate Program in Engineering of Processes and Technologies (PGEPROTEC), University of Caxias do Sul (UCS), R. Francisco Getúlio Vargas, 1130, 95070-560 Caxias do Sul, RS, Brazil; ‡ Department of Aerospace Structures and Materials, Faculty of Aerospace Engineering, 2860 Delft University of Technology, 2629 HS Delft, The Netherlands; § Department of Forestry Enginnering, Midwestern Parana State University (UNICENTRO), Rua Professora Maria Roza Zanon de Almeida, 84505-677 Irati, PR Brazil; ∥ Chaire Industrielle de Recherche Sur la Construction Écoresponsable en Bois (CIRCERB), Université Laval, 2425 Rue de la Terrasse, G1V 0A6 Québec (QC), Canada

## Abstract

The aim of this research is to evaluate the physical, chemical, thermal, and mechanical properties of Itauba (*Mezilaurus itauba*) wood fiber and Itauba wooden board. The chemical composition presented 33, 29, and 10% lignin, cellulose, and hemicellulose, respectively. The thermal stability was found to be 250 °C for both atmospheres (air and nitrogen), and the simulated TG curve was similar to the one performed on a nitrogen atmosphere. Cone calorimetric results showed a higher steady state when compared to other wood fibers found in the literature with peak heat release rates of 281.762, 424.029, and 482.335 kW/m^2^ when exposed to constant levels of radiant heat flux of 25, 50, and 75 kW/m^2^ at similar weights and densities. Furthermore, X-ray diffraction (13.5% crystallinity) and mechanical tests (flexural and tensile Young’s modulus of 12010 and 969.9 MPa, respectively) were performed on the Itauba wooden board. The tensile results showed to be higher than propylene composites reinforced with 40% wood fiber found in the literature while the storage modulus obtained in the dynamic mechanical thermal analysis found to be higher (11.5 GPa at −130 °C) than most of the commercial thermoplastics used in the industry (polypropylene (9 × 10^2^ MPa), high-density polyethylene (2 × 10^3^ MPa), and polyvinyl chloride (3000 MPa)). This study showed the potential in using Itauba wooden boards in replacing many commercial products, mainly when an adequate mechanical performance is required.

## Graphical abstract

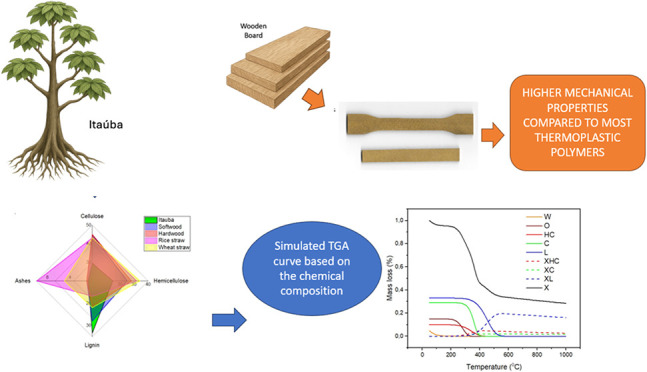

## 1 Introduction

Wood, primarily composed of cellulose, is the most abundant sustainable material on the planet, and along with its key component cellulose, it plays a crucial role in addressing significant societal issues.^1–3^ Although both cellulose and wood are carbon neutral, achieving a sustainable use requires advances within the wood industry. The pulp sector has made substantial progress in refining isolation processes in response to environmental regulations; however, there remains a pressing need for improved methods to safeguard biodiversity in both forests and agricultural areas.^4–6^ Utilizing agricultural waste as a cellulose source presents a partial solution. Managing a complex challenge demands extensive interdisciplinary research and development, which is often hindered by the current structure of academia. A transformation toward a more collaborative environment, rather than a tribal-like approach, is essential for obtaining such cooperation.^7,8^

New cellulosic materials have been characterized in the last decades while others have been discovered.^8–10^ Essentially, cellulosic materials are composed of cellulose, hemicellulose, and lignin. The amount of each component is dependent on several factors such as plant age, cultivation, and part of the plant (reason why literature points out a wide range of mechanical properties, for example). Cellulose is being applied in biomedical applications as tissue engineering, wound dressing, drug delivery, metal adsorption, biosensors, antioxidant, enzyme immobilization, among others.^11,12^ Hemicellulose-based materials can be used as sensors, adsorption, supercapacitors, packaging, lightning-controlled release, 3D-printing, among others.^13^ Lignin is generated as a waste product from the paper and ethanol production, stringent regulations for dust control, demand for high-quality concrete admixtures and dispersants, and carbon-rich products.^14^ For each mentioned example above, new extraction techniques and characterization have been studied, which increase the added value of each component of the wood fiber. Hence, the characterization and cataloging of new cellulosic materials (as Itauba fiber) are crucial both from the scientific point of view and as a source of valuable resources as income for local people.

Itauba wood fiber (*M. lindaviana*) is a potential wood Brazilian fiber from the North region of Brazil (Amazon and Brazilian Cerrado) (tropical savannah in eastern Brazil) distributed in Amazônia, Pará, Roraima, and Amapá states.^15–17^ Viana et al.^18^ evaluated the bond-line strength and chemical changes of Pine and Itauba joints welded by rotary friction and the use of the internal standard method with Rietveld refinement for the determination of the absolute cellulose crystallinity of wood samples. The specimens consisted of Itauba dowels fused into substrates made from both Pine and Itauba wood. The shear force resulting from the tensile pullout of the dowels was assessed through mechanical tensile testing. Additionally, a macrostructural analysis of the specimens was conducted alongside attenuated total reflectance-Fourier transform infrared spectroscopy (ATR-FTIR) and X-ray diffraction (XRD) examinations. The cellulose crystallinity was quantitatively evaluated using the internal standard method with Rietveld refinement and was compared against the Segal and deconvolution qualitative methods. The findings indicate that Itauba dowels welded into Itauba substrates yield stronger joints (0.81 MPa) and exhibit fewer chemical alterations compared to those in pine substrates (0.62 MPa). According to the authors, fewer chemical alterations can be attributed to the growth process. Itauba has a slower growth, and hence, Itauba has a narrow and almost indistinct earlywood and latewood. On the other hand, pine has fast growth and more distinct and wider earlywood and latewood. This large variation between earlywood and latewood combined with low density can lead to a more irregular and malformed welded bond line (with defects), similar to finger joints. The welded bond line between the Itauba dowel and the pine substrate demonstrates a higher cellulose crystallinity than that observed in the bond line with the Itauba substrate due to the reduction of some functional groups such as O–H, C–H, C=O, C–O, C + C, and C–O–C, as observed on FTIR analysis. This reduction was caused by the wood polymer degradation during the rotary friction welding process. Additionally, a less intense absorption band was observed for the 3800–2600 cm^–1^ region due to the dehydration effects caused by heating. This promoted oxidation and hydrolysis of acetyl groups in hemicelluloses and the modification of cellulose crystallinity. Another important factor is that the greater amount of hydrogen bonds with higher intensity among neighboring cellulose chains in Itauba slows heat transfer throughout wood and, consequently, its thermal degradation process. While the cellulose crystallinity shows qualitative consistency across the three methods employed, there are notable quantitative discrepancies among them. Rodrigues et al.^19^ focused this study on the technological characterization, machining, and processing of Itauba. The study assessed the technical properties and potential applications of Itauba wood sourced from two separate commercial lots by examining its mechanical properties and conducting machining tests. The authors performed static bending, dynamic, and machining tests. The perpendicular compression tests of the analyzed fibers yielded average values of 9899.77 and 10670.74 MPa for the elasticity modulus of lot 1 (L1) and lot 2 (L2), respectively. The average rupture modulus for L1 was recorded at 96.02 MPa, while that for L2 exhibited a value of 113.85 MPa. The shear tests revealed minimal variation among the specimens and between the lots evaluated. Regarding machining tests, Itauba wood was considered excellent for the production of furniture and internal openings, demonstrating acceptable mechanical strength, and its density is suitable for structural applications, as well as for the manufacture of furniture and interior elements.

There are a few studies found in the literature regarding technological applications of Itauba wood fiber, but no studies were found about the complete physical, chemical, thermal, and structural properties of Itauba powder and Itauba wooden board. Brazil has several forest species that can be used for sustainable wood production, and hence, the main objective of this study is to characterize Itauba wood fiber for potential utilization in composite materials, as well as to characterize Itauba wooden board fabricated 100% from Itauba tree.

## 2 Materials and Methods

### 2.1 Materials

Itauba wood was studied in powder and wooden board formats without any prior treatment. These woods were exclusively extracted from the heartwood of the tree. The raw material consisted of commercial boards supplied by Madeireira Bianchi, a local lumberyard in Bento Gonçalves, RS, Brazil. The process of machining and subsequent analysis is depicted in a schematic form in Figure 1. For mechanical testing, specimens were machined from the boards using a computer numerical control (CNC) system (Jaraguá, SC, Brazil), with the cutting direction aligned along the wood fibers. Prior to all analyses, both powder and board samples were oven-dried at 105 °C for 4 h to eliminate residual moisture.

**Figure 1:**
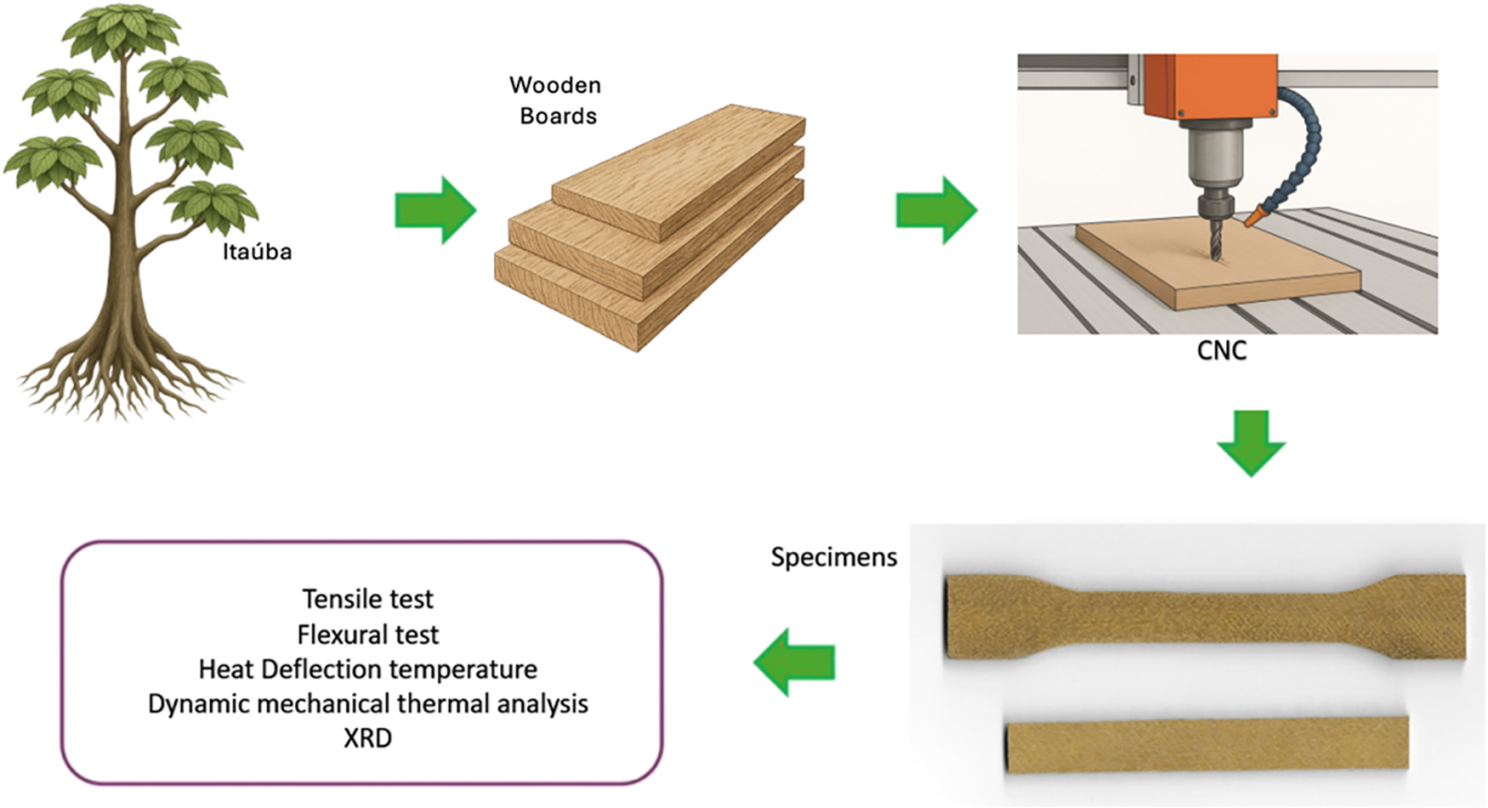
Schematic representation of the obtaining process of the specimens for the characterization of the Itauba wooden board (the first three illustrations were generated with AI assistance).

The raw wood was obtained, and the specimens for the mechanical, dynamic mechanical thermal analysis, heat deflection temperature, and X-ray diffraction were obtained using a CNC machine. For flexural and tensile tests, specimens were machined in accordance with the dimensional requirements of ASTM D790 and D638, respectively. For cone calorimeter analysis, samples were prepared with dimensions of 100 × 100 × 3 mm^3^. DMTA specimens measured 35 × 12 × 3.8 mm^3^. For heat deflection temperature (HDT) testing, specimens were prepared in accordance with the ASTM D648 standard dimensions.

### 2.2 Methods

The chemical composition was determined using the following methods: extractives (TAPPI T204 cm-97 ethanol/benzene solution at 1:2 proportion), lignin (TAPPI T222 om-02 Klason lignin), humidity, cellulose, and hemicellulose (Van Soest modified test).

The density of the specimens was determined according to ASTM 792 in ethanol.

FTIR spectra were obtained by means of a Nicolet IS10 spectrometer (Thermo Scientific, Waltham, MA). Natural fiber powder samples of each species (5 mg) were dispersed in a matrix of KBr (100 mg), followed by compression to form pellets. The sample collection was obtained using 32 scans, in the range of 4000 to 400 cm^–1^, at a resolution of 4 cm^–1^. Three different measurements for each fiber were evaluated, and the average value was considered.

X-ray diffractograms were collected using a sample holder mounted on a XRD-6000 diffractometer (Shimadzu, Kyoto, Japan) with monochromatic Cu Kα radiation (λ = 0.15418 nm). The generator was utilized at 40 kV and 30 mA, and the intensities were measured in the range of 5° < 2θ < 30°, typically with scan steps of 0.05° at 2 s/step (1.5°/min).

The thermogravimetric analysis was carried out on a TGA50 analyzer (Shimadzu, Kyoto, Japan) under constant nitrogen flow (50 mL/min), from 25 to 800 °C, at a heating rate of 10 °C/min. Approximately 10 mg of each sample was used. To simulate thermogravimetric curves based on chemical composition, we referenced a prior study conducted by Andrade et al.^20^ In summary, the authors based on a study suggested an initial composition for the samples that included hemicellulose, cellulose, lignin (Turku), lignin (alkaline), unextracted seeds, extracted seeds, and 11 hydrolyzed seeds, while also accounting for varying contents of water, oil, hemicellulose, cellulose, lignin, char (hemicellulose), char (cellulose), char (lignin), and inert materials for each sample. The characterization of the biomass was assessed through sugar content and solid analyses. Following the TGA of each component to validate the procedure for analyzing the effects of composition, structure, heating rate, and isothermal conditions, a mathematical model was formulated, which is regarded as a structural model in which cellulose is linked to lignin and hemicellulose. The reaction pathway adheres to the Waterloo mechanism, and the model is based on three distinct assumptions: (i) the reactions are irreversible and independent, (ii) all biomass components are maintained at a uniform temperature, and (iii) diffusional mass transport resistances for liquid phases are considered negligible. The mass balance for each component was nonstationary, with mass variations resulting from the reaction kinetics of the solid material and mass transfer of the liquid phases. The system was resolved using the eighth Runge–Kutta method and was validated through the Simplex Nelder–Mead and Broyden–Fletcher–Goldfarb–Shanno methods. The developed program (Free available Excel file) was used to perform the estimation of the TGA curves based on the chemical composition.

The materials were conditioned, prepared, and tested by using a dual-cone calorimeter supplied by Fire Testing Technology. The cone calorimeter was calibrated in accordance with ISO 5660–1.^21^ The sample surface dimension of 100 × 100 mm^2^ was exposed to constant levels of radiant heat flux of 25, 50, and 75 kW/m^2^, in a horizontal position, at 25 mm below the conical heating element. For data analysis, the following variables were evaluated: time to ignition (t_ig_), heat release rate (HRR), peak heat release rate (HR*R*_max_), total heat release (THR), mass loss (ML), and total smoke production (TSP).

Flexural tests were carried out according to ASTM D790 using an EMIC DL 3000 testing machine. Tensile tests were carried out according to ASTM D638 using an EMIC DL 3000 testing machine. Five specimens were tested for each tested, and the average values are reported. Dynamic mechanical thermal analysis (DMTA) was performed on rectangular specimens by using a Dynamic Mechanical Analyzer DMA2980. Analysis of the specimens (dimensions: 35 × 12 × 3.8 mm^3^) was performed in dual cantilever mode (oscillation amplitude: 15 lm) at 1 Hz frequency from −150 to 150 °C (heating rate of 3 °C/min). Heat deflection temperature (HDT) was carried out according to ASTM D648 using three distinct specimens.

## 3 Results and Discussion

### 3.1 Chemical Composition of Itauba Wood Fiber

The chemical composition of the Itauba fiber is presented in Table 1. Cellulose, hemicellulose, and lignin represent approximately 73 wt % of the wood fiber, while other components such as ashes, humidity, and extractives represent almost 27 wt % of the biomass.

**Table 1:** Chemical Composition of the Itauba Fiber Presenting the Humidity, Extractives, Cellulose, Hemicellulose, Lignin, and Ashes Content

humidity (% m/m)	extractives^a^ (% m/m)	cellulose (% m/m)	hemicellulose (% m/m)	lignin^b^ (% m/m)	ashes (% m/m)
11.06 ± 0.22	14.67 ± 1.42	29.50 ± 3.87	10.00 ± 2.40	33.33 ± 6.31	0.73 ± 0.11

a Extractives in ethanol/benzene solution 1:2.

b Lignin content considers all material that does not digest in a 72% p/p H_2_SO_4_ solution.

The results presented in Table 1 demonstrate that the lignin content was comparable to that of cellulose and about three times higher than the hemicellulose content.

Most lignocellulosic fibers found in the literature contain a higher cellulose content, which is the main crystalline component of the wood fiber. Cellulose is widely used in many fields such as papermaking, clothing, pharmaceuticals, and textiles, among others. Lignin is amorphous and responsible for major biorefining operations as an alternative to petroleum-based materials. Lignin can be burned to generate heat and sometimes power, be left intact to lend rigidity to the wooden constructs, and to convert biomass into ethanol and other liquid fuels (major biorefining operations).^22–24^ Hemicellulose acts as a binder between cellulose and lignin, contributing to the overall structural integrity and strength of plant tissues. On the other hand, hemicellulose is one of the most hygroscopic components of wood fiber, significantly influencing wood properties and wood–water interactions. There are numerous sorption sites, such as hydroxyl groups, that can engage with water molecules through the formation or disruption of hydrogen bonds. Furthermore, the naturally porous structure facilitates pathways for water molecules to enter or exit the wood. The chemical composition and hierarchical structure create the cellular environment for wood–water interactions, which influences both short- and long-term environmental performance.^23^

Figure 2 represents the radar plot comparing the main components of Itauba wood fiber with other wood fibers found in the literature. In general, the values are lower for Itauba compared to the other wood fibers, with the exception of lignin. Hemicellulose is about 3 times lower than other wood fibers found in the literature, while cellulose is about 25% lower.

**Figure 2:**
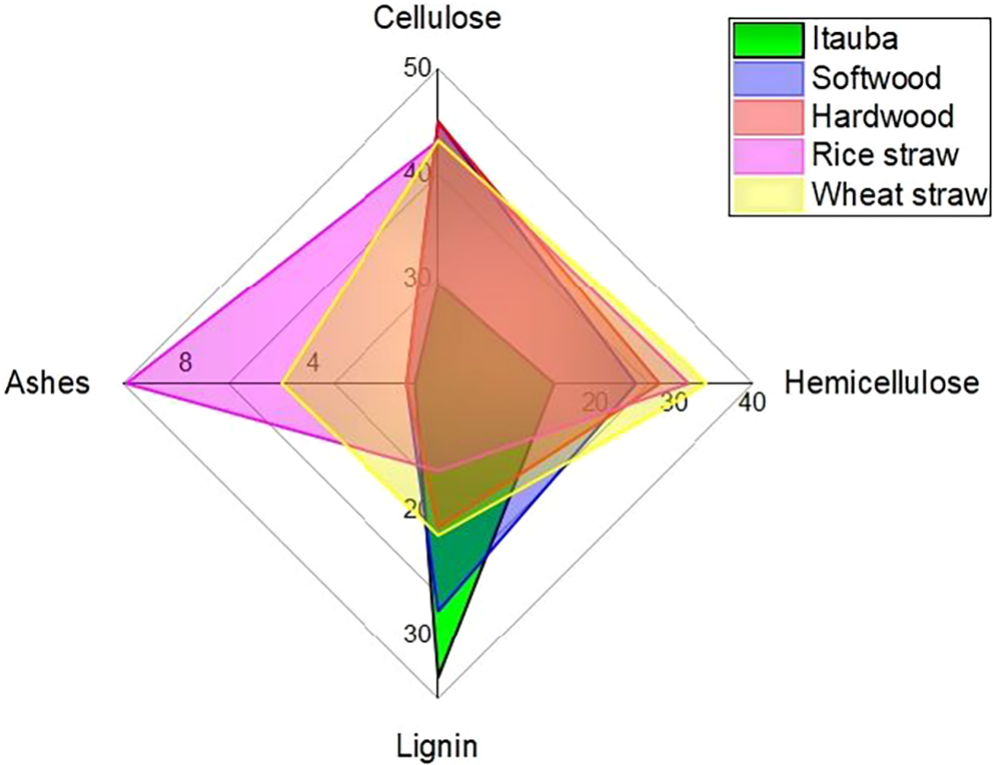
Radar plot of the comparison of Itauba wood fiber (green label) with other wood fibers found in the literature (softwood, hardwood, rice straw, and wheat straw are represented by blue, red, purple, and yellow labels, respectively). Adapted from data published in Pereira et al. (2015).

### 3.2 Physical and Chemical Characterization of Itauba Wood Fiber

The density of the Itauba wood fiber was 0.875 g/cm^3^ ± 0.02. This value is lower when compared to bast, bark, or stem fibers as flax, hemp, and banana or leaf fibers as pineapple or curaua (ρ = 1.5 g.cm^–3^).^26^ If incorporated as reinforcement in composite materials, lower density means a lightweight final product, which is excellent to save weight, for example. The lower hemicellulose amount (as observed in the chemical composition analysis) is also indicative of lower water absorption.

Fourier-transformed infrared spectroscopy (FTIR) was evaluated, aiming to detect the main absorption bands, as demonstrated in Figure 3. A prominent broad band is evident around 3400 cm^–1^, which is attributed to various O–H stretching modes. Additionally, two bands are observed at approximately 2920 and 2850 cm–^–1^, corresponding to the asymmetric and symmetric stretching of methyl and methylene groups, particularly in cellulose.^27^ Certain organic extractives, including fatty acid methyl esters and phenolic acid methyl esters, possess methyl and methylene groups.^27,28^ In the fingerprint region, bands at 1595, 1510, and 1270 cm^–1^ are associated with C=C and C–O stretching or bending vibrations from various lignin groups.^29^ Furthermore, the bands at 1460, 1425, 1335, 1220, and 1110 cm^–1^ are indicative of C–H and C–O deformation, bending, or stretching vibrations from multiple groups in lignin and carbohydrates.^27–29^ Finally, the bands at 1735, 1375, 1240, 1165, 1060, and 1030 cm^–1^are linked to C=O, C–H, C–O–C, and C–O deformation or stretching vibrations of various carbohydrate groups.^27–29^

**Figure 3:**
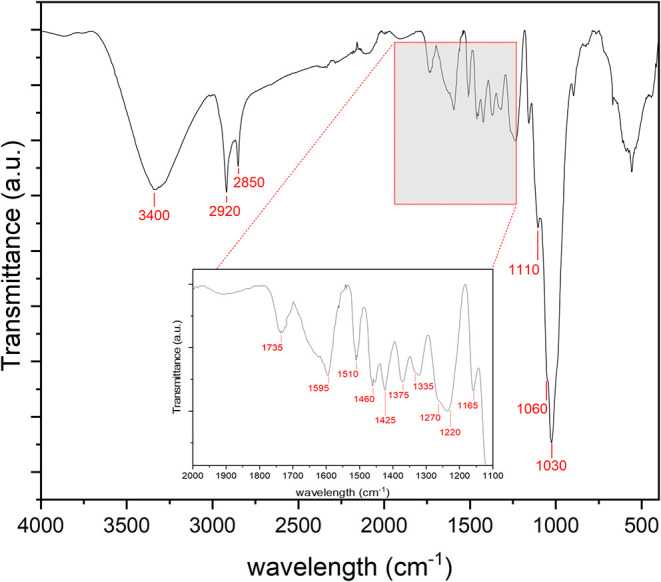
FTIR of the Itauba fiber. The main peaks are represented by numbers and/or a red square.

### 3.3 Thermal Properties of the Itauba Wood Fiber

Thermal degradation behavior of the Itauba fiber was studied in both air and nitrogen atmospheres (Figure 4). Independent of the atmosphere studied, the thermal degradation behavior occurred in three distinct stages: (i) an initial plateau from the start temperature until approximately 250 °C, (ii) an abrupt weight loss in a short time interval, and (iii) a new plateau until the final temperature test. There are some differences were both atmospheres are compared: on stage I, a similar behavior is observed for both atmospheres; on stage II, a faster weight loss is observed for air compared to nitrogen atmosphere, and finally on stage III (at approximately 400 °C), a continuously weight loss is observed at air atmosphere (until 700 °C, where no residual mass is observed, while on nitrogen atmosphere, slight weight loss is observed until 800 °C with ∼20% wt. residual mass loss). This is due to the oxidant atmosphere that speeds up the degradation of some components of the Itauba fiber. The simulated curve (further explained) follows the nitrogen curve behavior.

**Figure 4:**
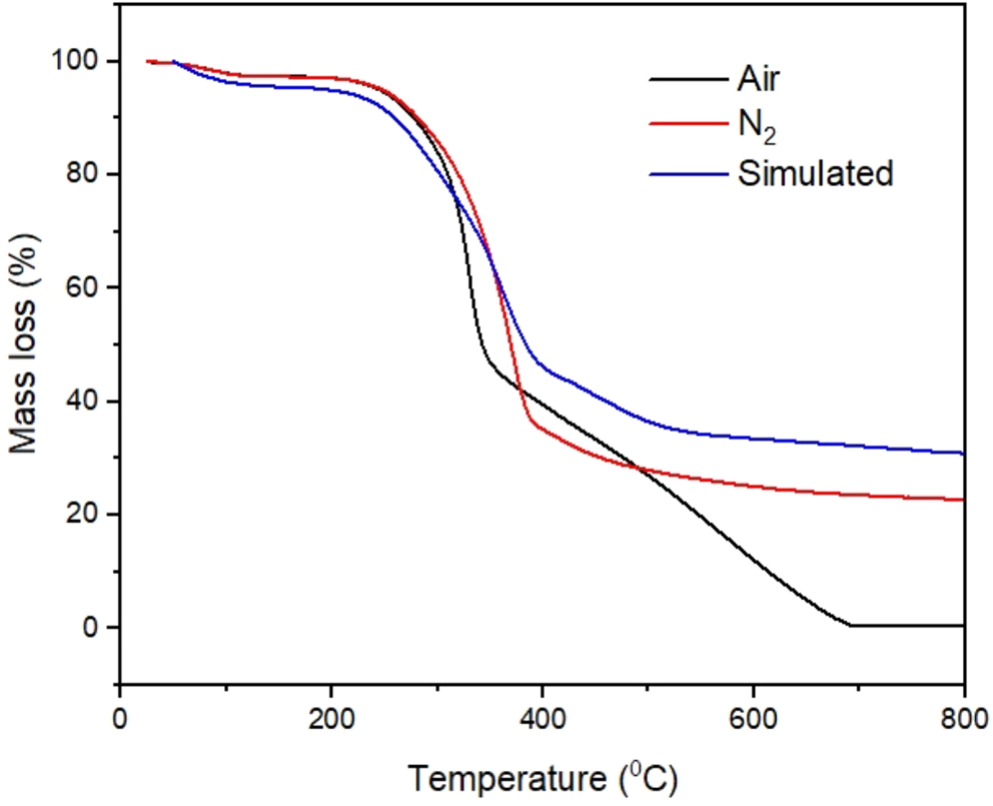
Thermal degradation behavior of Itauba wood fiber using air (black line) and nitrogen atmosphere (red line) and simulated curve (blue curve). The simulated curve was done according to ref^20^ and properly described in the Materials and Methods Section.

The TG behavior can be better explained by understanding the vegetal fiber structure (Figure 5).^30–35^ Cellulosic materials are mainly composed of cellulose, hemicellulose, and lignin (intrinsic water and waxes are also presented), which are responsible for the mechanical, physical, and chemical properties of the fibers. It is noteworthy to mention that not only the amount of the components is important but also the way they connect to each other. That is the reason there are many variations in the properties even considering similar chemical composition proportions.

**Figure 5:**
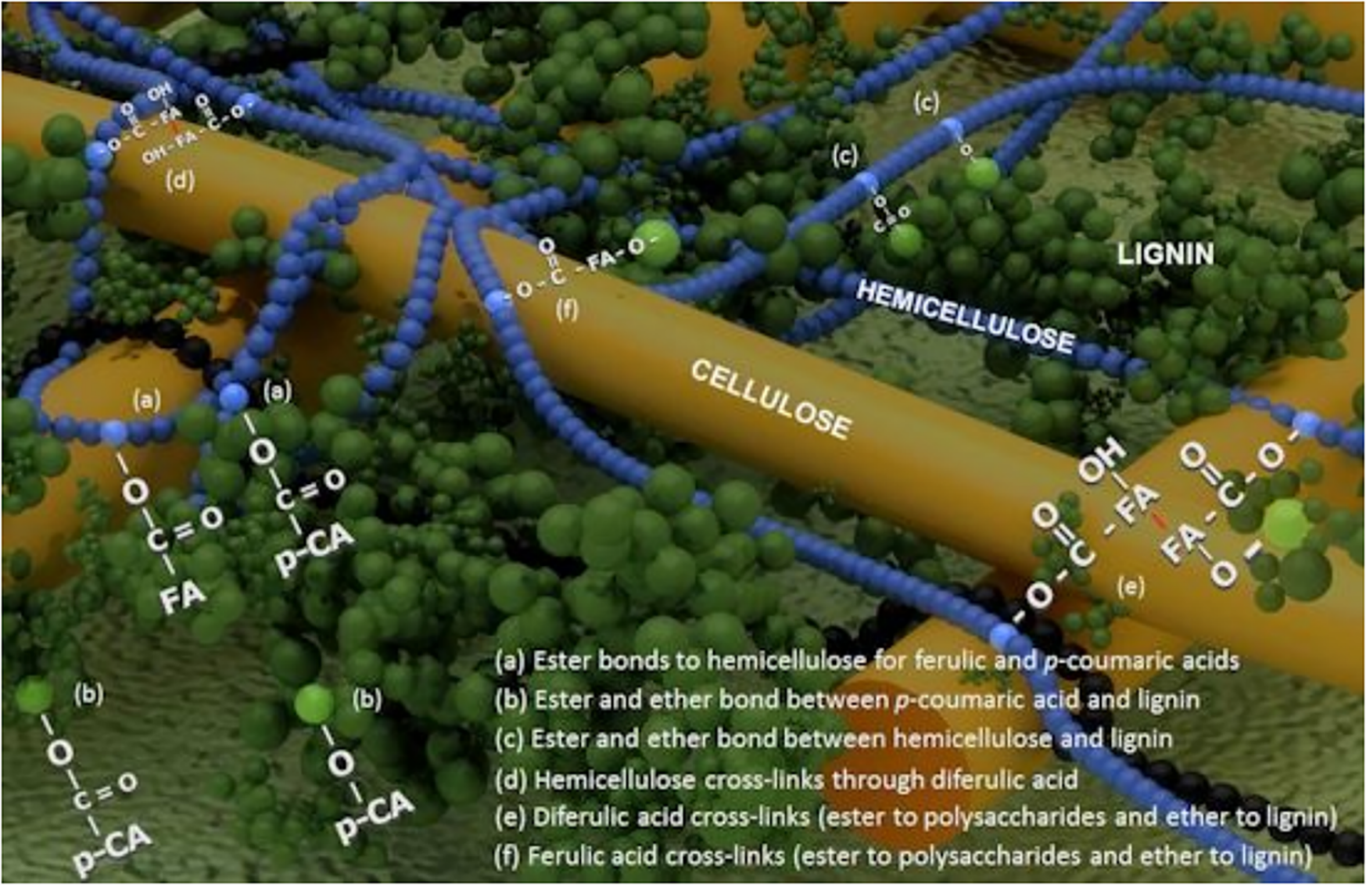
Detailing of the interaction among cellulose, hemicellulose, and lignin. Adapted from [34], used under the terms of the Creative Commons license. Different interactions (a–f) and bonds among the components.

Based on the chemical composition, a thermogravimetric curve was simulated (Figure 6), where it is possible to observe the individual behavior of cellulose, hemicellulose, lignin, extractives, and water on the main degradation curve.^35,36^ The respective effects of the cellulose, hemicellulose, and lignin volatiles are also observed. Cellulose exhibited a relatively rapid decomposition, with the majority of weight loss occurring between 250 and 400 °C. The same behavior was observed when hemicellulose was analyzed. The residual mass loss for both components is nearly 2% (observed in hemicellulose and cellulose charcoal). Among the three components, lignin proved to be the most resistant to decomposition. Its degradation was gradual across the entire temperature spectrum being the most notable degradation step occurring in the temperature range from 400 to 520 °C. The solid residue remaining after lignin pyrolysis was the highest, at 20 wt %.^32^ Hence, it seems clear that the thermal decomposition follows the order: hemicellulose/cellulose and lignin. However, this decomposition is overlapped in certain temperature ranges, mainly for hemicellulose and cellulose. Lignin decomposes across all temperature ranges, and it is responsible for the main residual mass loss of the vegetal fiber. Previous studies from the literature indicated that vegetal fibers containing more cellulose content tend to result in composites exhibiting higher tensile or flexural resistance. Other studies demonstrate that the fiber content is more important for the mechanical properties than the fiber composition.^25,33^ The reason is that the number of interactions among the three major components is considerable, as is their complexity. That is the reason why the mechanical property of a composite material is difficult to estimate only by the amount of cellulose or hemicellulose content of the fiber but essentially by the filler amount, matrix/filler interaction (adhesion), and chemical affinity, among other factors. Another issue is that the former decomposition of one of the components can generate volatiles that can accelerate the decomposition of the remaining structure. Independently of the structure of the wood, it is very important to determine the cell wall composition and how it can interact in different fields, as modifications of crops to withstand pests and diseases,^34^ biomedical fields,^11^ or as reinforcement in composite polymeric materials.^35–37^ Since wood has been extensively utilized in human life for different purposes, wood has also been transformed into advanced functional materials, including transparent wood, shape-memory wood, and phase-change storage wood. By knowing that wood is inherently hygroscopic, in everyday applications, the moisture present in the surrounding environment continuously interacts with wood, inevitably resulting in dimensional instability and potentially leading to a decline in mechanical properties and durability. Consequently, the service value and lifespan of wood products and wood-based functional materials are considerably affected. To optimize the utilization of wood-based materials, it is crucial to undertake comprehensive research on the interactions between wood and water.^23^

**Figure 6:**
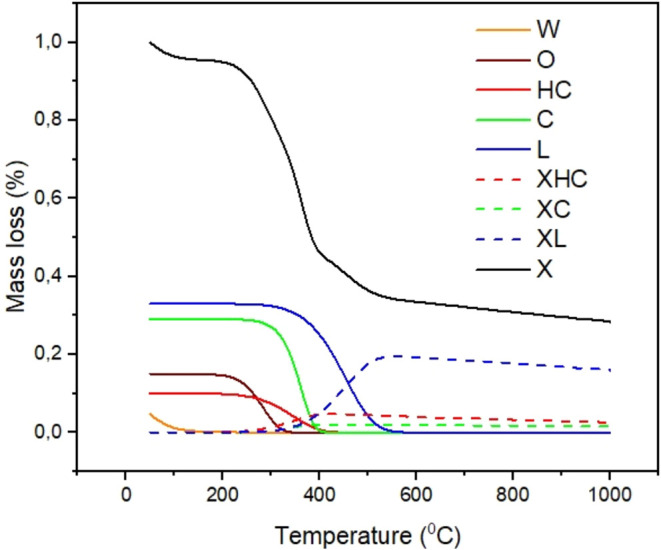
Simulated TG curve based on the chemical composition of the Itauba fiber. W – water (continuous yellow curve), O – oil (continuous brown curve), HC – hemicellulose (continuous red curve), C – cellulose (continuous green curve), L – lignin (continuous blue curve), XHC – hemicellulose charcoal (dashed green curve), XC – cellulose charcoal (dashed red curve), XL – lignin charcoal (dashed blue curve), and X – entire curve (continuous black curve).

### 3.4 Cone Calorimeter Tests

Table 2 presents the values obtained for the variables analyzed in the calorimetric cone tests. The sample surface dimension of 100 × 100 mm^2^ was exposed to constant levels of radiant heat flux of 25, 50, and 75 kW/m^2^ at similar weights and densities. The total heat release (THR), peak heat release rate (HR*R*_max_), and total smoke production (TSP) are also included for the three distinct heat fluxes. The heat release rate (HRR) represents the amount of heat released during combustion between the fuel and the oxidizer under the effect of a heat source. HRR is determined in the cone calorimeter based on oxygen consumption during combustion and is expressed in kW/m^2^, and the total heat energy produced represents the total heat release (THR). The heat release rate peak (HR*R*_max_) represents the maximum value of the HRR curve. TSP (total smoke production, in m^2^) is calculated based on the production of carbon monoxide and carbon dioxide, combined with the smoke extinction coefficient measured in the exhaust duct of the cone calorimeter using a laser integrated into the equipment. These parameters are crucial to assess, as the greatest hazards during a fire come from smoke and combustion products.^21,38^ The results were presented in the form of tables and graphs. It is observed that all parameters increase with the heat flux, which is expected.

**Table 2:** Averages Were Calculated for the Calorimetric Cone Test Variables^a^

	incident heat flux (kW/m^2^)		
parameter	mean25	mean50	mean75
weight (g)	42.128	42.698	43.410
thick (mm)	4.709	4.895	4.879
density (g/cm^3^)	0.901	0.873	0.890
peak heat release rate (HR*R*_max_) (kW/m^2^)	281.762	424.029	482.335
total heat release (THR) (MJ/m^2^)	45.936	55.502	54.868
total smoke production (TSP) (m^2^)	2.506	2.585	2.780

a The weight, thick, and density of the Itauba fiber are presented for different incident heat fluxes. Also, the peak heat release rate, total heat release, and total smoke production are presented.

Figure 7 represents the heat release rate (HRR) chart as a function of time for the different incident heat fluxes in the test samples. The total heat release (THR) is similar for all heat fluxes, indicating that the energy conservation is practically constant. The HRR peak increases and shifts to a lower time with the heat flux. As a consequence, the mass loss occurs faster with the heat flux (Figure 8). The mass loss rate (MLR) (shown in Figure 8), expressed in grams per second (g/s), refers to the rate at which the material loses mass due to thermal degradation and combustion. The mass loss expresses the mass degradation of the materials. The mass loss represents the thermal degradation of the material, indicating the amount of mass that is consumed or released during combustion. Comparing the results obtained at 50 kW/m^–2^ with other wood fibers, a similar behavior was observed among different wood (red oak, sugar maple, Douglas fir, and white spruce) species during combustion.^38^ After ignition, there was a fast increase in the heat release rate, which then decreased and stabilized until a char layer formed. However, a second peak appeared at the end of the combustion of the Itauba wood fiber; the same behavior was observed for the red oak species.^38^ However, a steady state as high as for sugar maple species is observed for sugar maple.

**Figure 7:**
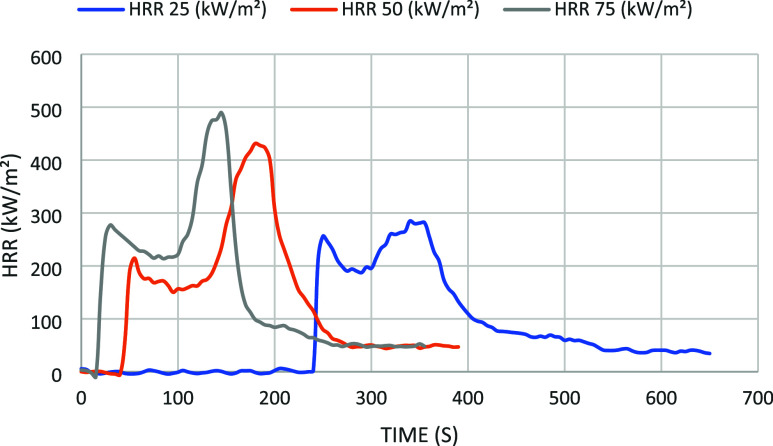
HRR chart as a function of time for the different incident heat fluxes in the test samples. The incident heat fluxes of 25, 50, and 75 kW/m^2^ are represented by the blue, orange, and gray lines, respectively.

**Figure 8:**
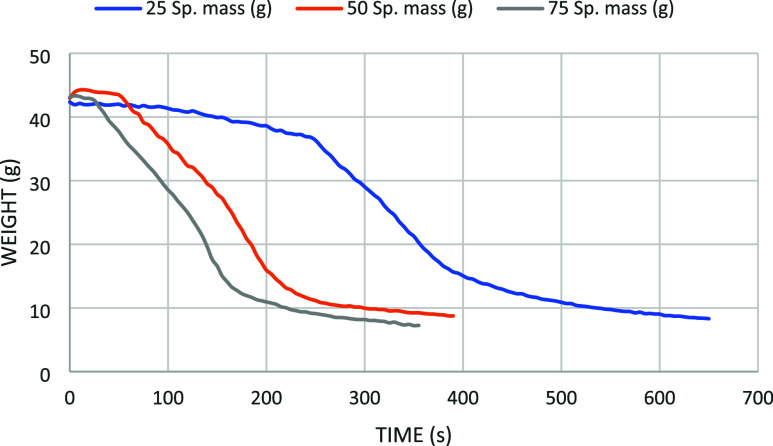
Mass loss chart as a function of time for samples submitted to each incident heat flux. The incident heat fluxes of 25, 50, and 75 kW/m^2^ are represented by the blue, orange, and gray lines, respectively.

### 3.5 X-ray Diffraction (XRD) of Itauba Wooden Board

XRD (Figure 9 A–C) of the Itauba wooden board was estimated, aiming to determine the crystallinity index of the Itauba wooden board. The crystallinity index estimated was 13.95% at 2Θ = 5–90°, according to the method used (peak deconvolution). The value is lower than the ones found in the literature. The crystallinity index of wood fibers lies in the range of 54.4–77%.^39^ We assume that the most controversial analysis is related to crystallinity index determination. One of the main reasons is the method used: the most prevalent is the Segal peak, followed by peak deconvolution and the Rietveld method. According to French,^40,41^ the assessment of the amorphous component using the Segal method is significantly affected by the overlap of crystalline peaks. In contrast, the peak deconvolution technique relies solely on observable peaks, which may result in the misattribution of some crystalline intensity to the amorphous component or the background. Furthermore, the Rietveld method involves numerous variable parameters and lacks a clear determination of the unique data points necessary for effective refinement. Briefly, the method used must be graphically represented by the authors to allow the results to be equally compared because the area calculated also involves amorphous cellulose. A great part of the papers that estimate vegetal fiber crystallinity uses the Segal’s method,^41^ recommending that the authors move beyond this method, which overestimates the values. This statement seems to be clear when the reader compares the cellulose content obtained in the chemical composition and the values obtained by XRD; i.e., the remaining cellulose is amorphous cellulose that contributes to the peak but not to the crystallinity. The data from Figure 2 shows cellulose content up to 50% for different wood fibers; the same fibers presented crystallinity indexes as high as 70% (shown at the beginning of this section). It seems that there are some discrepancies because the crystallinity index measured by XRD is higher than that determined by chemical composition. This would be explainable if only cellulose were measured and not the entire vegetal fiber (in this case, it would mean that 70% of cellulose is crystalline). Even so, almost all authors did not consider the contribution of the amorphous crystal to the area of the curve. In our opinion, any method to determine the crystallinity index is valid since it is properly described (indicating the mathematical procedure, deconvolution parameters, among others). The method also depends on the tools available to the researchers. For example, mathematically complicated methods (as Rietveld) can be more precise, but few researchers can be apt to perform them, while other methods can be dependent on the initial conditions input in the software (Segal method). It is important to properly describe the method used. We opt for the peak deconvolution method, in which all of the area below the curve is considered, avoiding arbitrariness in the choice of the initial parameters, as in the case of the Segal method (the high noise of the raw diffraction data makes the choice of one value for the maximum peak).

**Figure 9:**
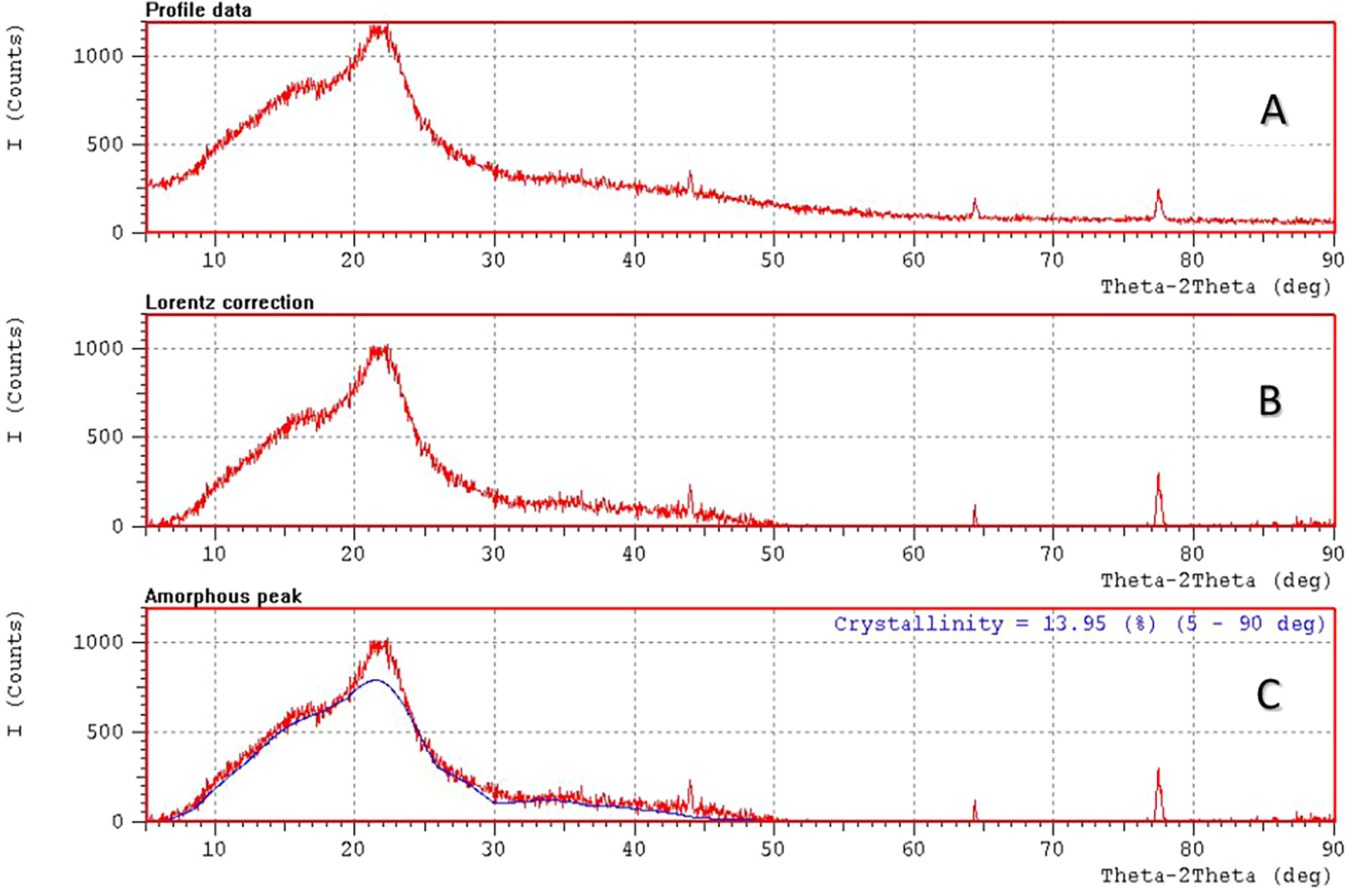
XRD curves of the Itauba wooden board. (A) Raw data, (B) Lorentz correction, and (C) crystallinity index considering the peak deconvolution method (fitting is represented by the blue line).

### 3.6 Mechanical Resistance of Itauba Wooden Board

The heat deflection temperature (HDT) of the Itauba wooden board showed a value of 0.186 ± 0.01, while the tensile and flexural results are presented in Table 3. The values found for Itauba are higher compared to polypropylene composites filled with 40% wood flour (3610 MPa for 70 mesh size wood) and flexural modulus (3150 MPa for 70 mesh size wood). Lower elongation was also observed for the composite (2.27% for 70% mesh size wood) compared to 7.828% for Itauba wood fiber.^42^ If compared to Chinese fir (*C. lanciolata*), the values are lower (around 23000 MPa against 968.9 for Itauba wood fiber). The elongation at break for Chinese fir was lower (3.5% for 35% lignin content) than 7.8% for Itauba wood fiber.^43^

**Table 3:** Flexural and Tensile Mechanical (Modulus of Elasticity and Elongation at Break) Results for the Itauba Wooden Board

test	modulus of elasticity (MPa)	elongation at break (%)
flexural	12010 ± 1906	1.501 ± 0.23
tensile	968.9 ± 119.8	7.828 ± 1.848

The dynamic mechanical thermal analysis (DMTA) curves of the Itauba wooden board are presented in Figure 10. The storage modulus (black curve) shows an initial value of around 11.5 GPa at −130 °C, passing through the glass transition temperature (*T*_g_) at −100 °C (better observed in the loss modulus and tan δ peaks, represented by the red and blue curves, respectively). After *T*_g_, a rapid decrease in the storage modulus value is observed (8 GPa at 50 °C). Phenomenologically, this decreasing can be explained by a considerable increasing in the molecular mobility of the main backbone chain, which allows a higher number of conformation states for the same energy level.^44^ So, the energy storage in the system (more specifically in the main backbone) is dissipated as heat through reptation movement (similar to reptiles). This movement is more attenuated with more available spaces in the system, i.e., higher free volume hole. At last, if more space is found in the system, the energy storage capability is lowered, and consequently, the storage modulus value is decreased.^34,44^ The values obtained for the storage modulus in the glassy region are higher when compared to polypropylene (9 × 10^2^ MPa),^45^ high-density polyethylene (2 × 10^3^ MPa),^46^ and polyvinyl chloride (3000 MPa).^47^

**Figure 10:**
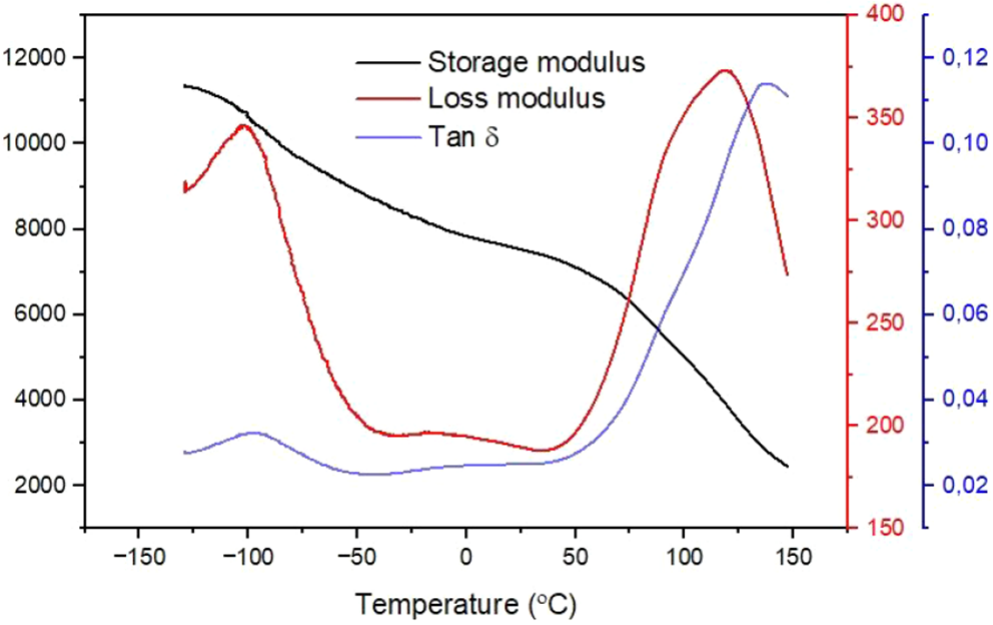
Dynamic mechanical thermal analysis of the Itauba wooden board. The storage modulus (black line), loss modulus (red line), and tan δ (blue line) are presented.

## 4 Conclusions

There are few studies in the literature about Itauba wood characterization and the potential use of Itauba wooden boards as a replacement for many polymers. Therefore, the main objective of this study is to characterize Itauba wood fiber as a potential utilization in composite materials, as well as to characterize the Itauba wooden board fabricated 100% from the Itauba tree. For the Itauba wood fiber, chemical composition, Fourier-transform infrared spectroscopy (FTIR), density, thermogravimetry (TGA), and cone calorimetric tests were evaluated. The chemical composition analysis showed a higher lignin content (33%) compared to other wood fibers found in the literature as rice (15%) and wheat (22%) straw. The thermogravimetric curve simulated using the chemical composition data showed similar results (for both air and nitrogen atmospheres), mainly in the thermal plateau. For the terminal region, the results are more similar when compared to nitrogen than to the air atmosphere. The thermogravimetric curve follows a similar pattern compared to other wood fibers found in the literature, indicating that the thermal stability is not dependent on an individual component but on the own wood fiber characteristics. The calorimetric test, similar results were performed at three distinct heating fluxes, but the results were compared at 50 kW.m^–2^ with data found in the literature for red oak, sugar maple, Douglas fir, and white spruce species during combustion. A similar curve pattern was found with red oak but a higher steady state than most of the species compared with the literature. X-ray diffraction (XRD) and mechanical tests were performed on an Itauba wooden board. XRD results showed a lower crystallinity index (14%) compared to other wood fibers from the literature (54.4–77% crystallinity) due to the high lignin content of Itauba wood. The mechanical tests showed that the flexural Young’s modulus is higher (12010 MPa) than some thermoplastic composites such as polypropylene composites filled with 40% wood flour (3610 MPa for 70 mesh size wood) found on literature, while the storage modulus determined by dynamic mechanical thermal analysis (DMTA) showed to be higher than most of the commercial thermoplastic found on industry (∼11.5 GPa in the glassy region for Itauba compared to some thermoplastics as polypropylene (9 × 10^2^ MPa), high-density polyethylene (2 × 10^3^ MPa), and polyvinyl chloride (3000 MPa)). The results demonstrate that the Itauba wooden board can be used as a replacement for many polymers as polypropylene, high-density polyethylene, and poly­(vinyl chloride), which can promote an ecofriendly and sustainable product. For future studies, it is suggested that the Itauba wooden board be studied in situ to simulate real applications. In this case, studies of accelerated aging and salt spray would be useful.
